# A Systematic Review of Screen Time and Obesity in Children, Adolescents, and Young Adults: Sex-Specific Pathways and Implications for Public Health Interventions

**DOI:** 10.5888/pcd23.260212

**Published:** 2026-10-15

**Authors:** Jinsoo Min, Yoomi Chae, Yoolwon Jeong

**Affiliations:** 1 Division of Pulmonology and Critical Care Medicine, Department of Internal Medicine, Seoul St. Mary’s Hospital, College of Medicine, The Catholic University of Korea, Seoul, Republic of Korea; 2 Department of Medical Education, College of Medicine, Dankook University, Cheonan-si, Republic of Korea; 3 Department of Preventive Medicine, College of Medicine, Ewha Womans University, Seoul, Republic of Korea

## Abstract

**Introduction:**

Screen time is associated with multiple adverse health outcomes, including obesity. However, evidence for children and adolescents is limited and inconsistent, and potential sex-specific differences have not been characterized. This systematic review aimed to synthesize evidence on the association between screen time and obesity-related outcomes among children, adolescents, and young adults, with a focus on sex-specific pathways and implications for public health interventions.

**Methods:**

This systematic review followed Preferred Reporting Items for Systematic Reviews and Meta-Analyses 2020 guidelines and was based on an a priori protocol. Two reviewers independently selected studies, extracted data, and assessed risk of bias using the Newcastle–Ottawa Scale. Data were synthesized based on the direction of associations and patterns of sex differences.

**Results:**

Of the 22 studies included, 18 reported a significant positive association between screen time and obesity-related outcomes. Sex-specific patterns were also observed, with some studies reporting associations only among females (n = 6) or males (n = 4). Among females, observed associations more frequently involved adiposity, metabolically unhealthy obesity, and psychologic factors such as body image concerns and weight dissatisfaction, whereas among males, associations were more often linked to behavioral factors such as increased caloric intake, late-night screen use, and reduced sleep.

**Conclusion:**

Our findings indicate that screen time is associated with obesity among children, adolescents, and young adults, with evidence suggesting potential sex differences in the behavioral, physiologic, and psychologic mechanisms. These findings highlight the need for interventions that move beyond simple screen time reduction and consider the mechanisms that may underlie sex-related differences in obesity risk. Potential strategies may include promoting physical activity among girls and addressing problematic screen use, screen-related eating, and sleep disruption among boys, as well as integrating screen-related psychosocial factors into obesity prevention and management. Given the limited number of studies formally assessing sex–screen time interactions, further research is needed to better characterize these observed patterns.

**Summary**

**What is already known on this topic?**

Excessive screen time has been associated with obesity and related health behaviors, including poor diet, reduced physical activity, and inadequate sleep. However, evidence for children and adolescents is limited, particularly regarding sex-specific differences, with few studies formally testing sex interactions.

**What is added by this report?**

Our findings indicate that screen time is consistently associated with obesity-related outcomes among children, adolescents, and young adults, with sex-specific pathways involving behavioral, physiologic, and psychologic mechanisms.

**What are the implications for public health practice?**

Screen time interventions should move beyond general reduction strategies to incorporate sex-specific approaches, including addressing behavioral patterns, sleep, and psychologic factors such as body image and weight concern.

## Introduction

Screen time has increased rapidly with the widespread adoption of smartphones and digital devices (1,2). A growing body of evidence suggests that excessive screen exposure is associated with a range of adverse health outcomes, including reduced physical activity, sleep disturbances, and mental health problems (3–6). In response, multiple public health and clinical guidelines have recommended limiting screen time among children and adolescents and promoting balanced use of digital media (7–9). Despite these recommendations, screen use continues to rise globally, highlighting the need to better understand its health effects and underlying mechanisms.

Among the health outcomes associated with screen time, obesity has emerged as a primary concern. Although evidence from adult populations has increasingly demonstrated a positive association between screen exposure and obesity-related outcomes (10–12), findings among children and adolescents are comparatively limited. This gap is particularly concerning given that children and adolescents may be more vulnerable to obesity-related health outcomes because of the developmental processes that occur during childhood and adolescence (7–9,13,14). In addition, sex differences in behavioral, physiologic, and psychosocial pathways suggest that the relationship between screen time and obesity may not be uniform across populations (15,16). Despite this evidence, relatively few studies have examined sex-specific associations or formally tested interactions between screen time and sex, resulting in an incomplete understanding of differential risk patterns.

The objective of this review was to systematically synthesize the existing evidence on the association between screen time and obesity-related outcomes among children, adolescents, and young adults, with a focus on sex-specific patterns, potential mechanisms, and implications for public health interventions.

## Methods

We conducted this systematic review in accordance with PRISMA (Preferred Reporting Items for Systematic Reviews and Meta-Analyses) 2020 guidelines, which informed the design, conduct, and reporting of the review (17). The review protocol was developed a priori and registered on the Open Science Framework (https://osf.io/y6gck), ensuring transparency in the review process. Ethical approval was not required because the study was based exclusively on previously published literature and did not involve individual-level human data. The study was conducted in accordance with the Bioethics and Safety Act (Act No. 15188) and its Enforcement Rule (Ordinance No. 1048) of the Ministry of Health and Welfare, Republic of Korea.

### Data sources

We conducted a comprehensive literature search from March 1 through March 15, 2026, across 3 electronic databases: CINAHL, Embase, and PubMed. We developed the search strategy to identify relevant studies using a combination of controlled vocabulary (eg, Medical Subject Headings and Emtree terms) and free-text keywords. Search terms included those related to screen-based behaviors (eg, screen time, television viewing, smartphone use, social media, video gaming), obesity-related outcomes (eg, obesity, overweight, body mass index [BMI], adiposity, body fat, fat mass), pediatric populations, including studies with age ranges extending into young adulthood (ie, children, adolescents, young adults), and sex-specific differences. We conducted citation tracking and snowball searching to identify additional studies. We restricted to studies published from 2015 onward to reflect contemporary patterns of screen use after the widespread adoption of smartphones and evolving public health guidelines.

### Study selection

We initially identified 1,342 records (Figure 1). Before screening, we pilot tested the process on a subset of 55 studies to ensure consistency between reviewers. During title and abstract screening, we excluded 524 records based on the predefined inclusion criteria. Eligible studies were those that 1) involved human participants, 2) assessed exposure to screen time, 3) reported obesity-related outcomes, 4) reported sex-specific results, including sex-stratified analyses and/or formal tests of sex differences, 5) focused on children, adolescents, and young adults, and 6) were published in English. To be eligible for inclusion, studies were required to report sex-specific effect estimates or formally examine sex differences through interaction analyses. We excluded studies that adjusted for sex as a covariate without presenting sex-specific results or testing interaction. We did not include gray literature (reports and other non–peer-reviewed sources) in our search.

**Figure 1:**
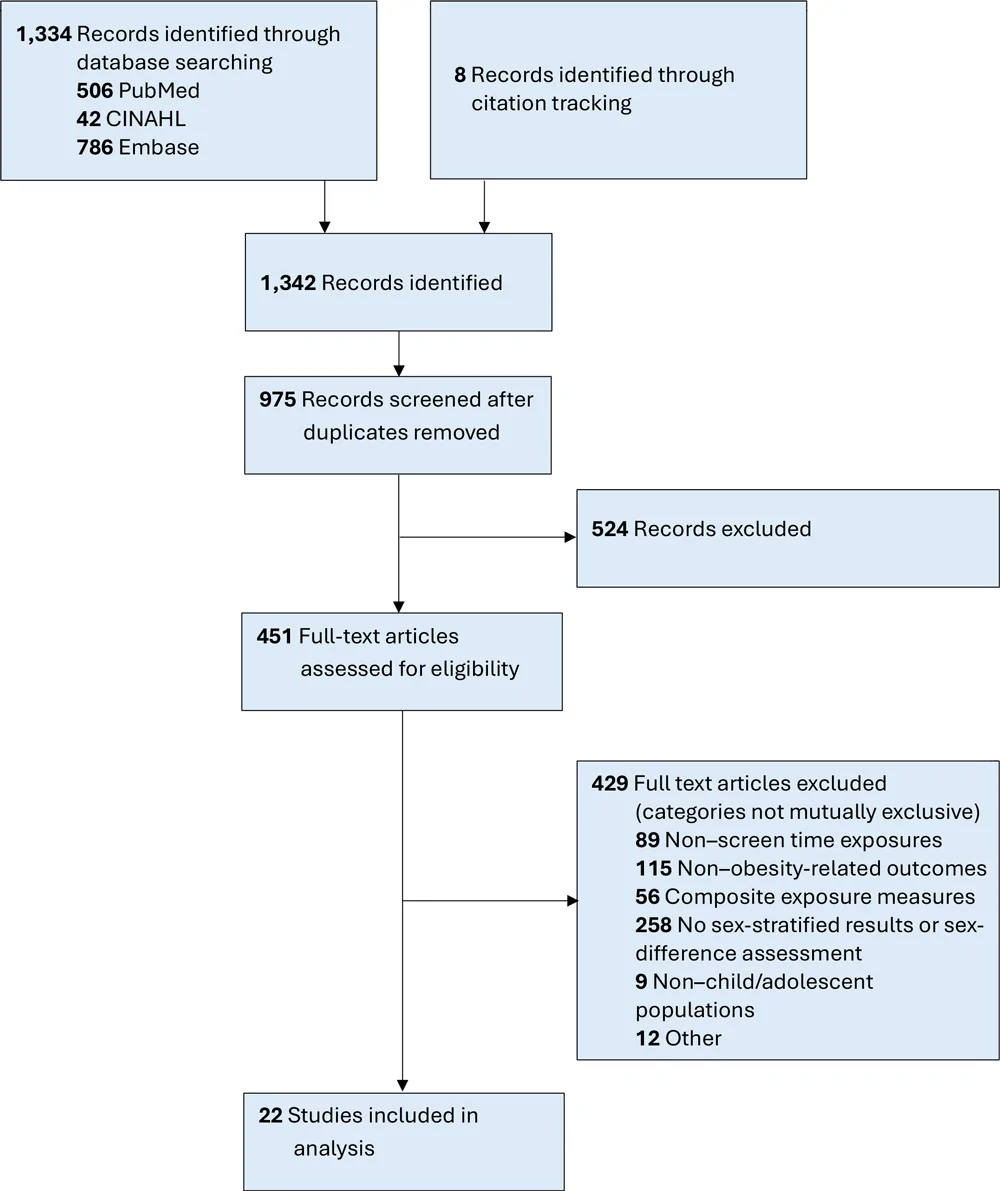
Flowchart outlining the study search and selection process in a systematic review of screen time and obesity among children, adolescents, and young adults, with a focus on sex-specific differences.

We assessed 451 full-text articles for eligibility. We excluded studies if they 1) did not examine screen time as an exposure and obesity-related measures as outcomes, 2) assessed screen time as part of a composite measure (eg, adherence to movement guidelines or physical activity recommendations), 3) did not conduct sex-specific analyses, or 4) did not study children or adolescents. Screening and study selection were conducted independently and in duplicate by 2 reviewers (J.M., Y.J.). We resolved discrepancies through discussion with a third reviewer (Y.C.). Two reviewers (J.M., Y.J.) independently used the Newcastle–Ottawa Scale (NOS) to assess risk of bias of the included studies. We predefined criteria for each NOS subscale based on the review objectives. Ultimately, we included 22 studies in the final review.

### Data extraction

Two reviewers (J.M., Y.J.) independently extracted data by using a pilot-tested standardized extraction form. Extracted data included study characteristics as well as exposure and outcome variables relevant to the review question. For screen time, we extracted information on how it was measured (eg, self-reported or objectively measured), specific types of screen time assessed (eg, television viewing, gaming, social media use, total screen time), and how the variables were operationalized for analysis (eg, categorical, continuous). For obesity-related outcomes, we extracted data on measurement methods (eg, direct measurement, record-based), and outcome definitions (eg, weight status classification, BMI-based measures such as BMI percentiles or *z* scores, waist-to-height ratio), and reference standards or criteria used.

Extracted data were synthesized by using a structured narrative approach and categorized according to the direction and significance of the association between screen time and obesity-related outcomes. We further classified studies according to sex-specific patterns of association (ie, associations observed only among males or females, both sexes, or neither sex), as well as whether formal tests for interaction were conducted. Two reviewers (J.M., Y.J.) extracted data independently in duplicate after a pilot phase to ensure consistency in the use of the extraction form. Given the substantial heterogeneity in exposure definitions, outcome measures, and study designs across studies, we did not perform a meta-analysis. Although a small subset of studies examined total screen time in relation to a common BMI-related outcome, differences in study design and analytical approaches limited the interpretability of pooled estimates. Accordingly, we considered a narrative synthesis to be the most appropriate format for summarizing the available evidence.

## Results

The 22 studies (18–39) were conducted across diverse geographic settings, including Australia (n = 2), China (n = 6), and the US (n = 5), among other countries, and 1 multicenter study conducted across several European countries (Table 1).

**Table 1:** Summary of Studies Examining the Association Between Screen Time and Obesity-Related Outcomes Among Children, Adolescents, and Young Adults, 2015–2026

Study	Country	Study population		Study design
		Age, y	No. of participants	
Al-Hazzaa and Albawardi (18), 2019	Saudi Arabia	15–19	2,888	Cross-sectional
Al-Shoaibi et al (19), 2026	US	9–11	3,498	Longitudinal (prospective cohort)
Cai et al (20), 2022	China	7–18	15,114	Cross-sectional
Domoff et al (21), 2021	US	13–16	193	Cross-sectional
Foubister et al (22), 2023	UK	14	10,798	Cross-sectional
Furthner et al (23), 2017	Austria	10, 14, 17	2,930	Cross-sectional
Golshevsky et al (24), 2020	Australia	4–17	343	Cross-sectional
Kenney and Gortmaker (25), 2017	US	14–18	24,800	Cross-sectional
Lu et al (26), 2025	China	6–20	47,148	Longitudinal (prospective cohort)
O’Brien et al (27), 2018	Ireland	12–14	169	Cross-sectional
Padmapriya et al (28), 2019	Singapore	2–5	956	Longitudinal (prospective cohort)
Parkes et al (29), 2020	UK (Scotland)	46–122 mo	2,810	Longitudinal (prospective cohort)
Ren et al (30), 2017	China	7.3–11.9	5,032	Cross-sectional
Saelee et al (31), 2020	US	11–19	12,692	Longitudinal (prospective cohort)
Sampasa-Kanyinga et al (32), 2020	Canada	11–20	4,991	Cross-sectional
Seral-Cortes et al (33), 2021	Europe^b^	12.5–17.5	2,047	Cross-sectional
Shakir et al (34), 2018	Australia	10–13	234	Cross-sectional
Sun et al (35), 2021	China	11.0–11.3	5,535	Longitudinal (prospective cohort)
Tan et al (36), 2025	China	12–15	438	Cross-sectional
Xue et al (37), 2016	China	7–15	1,586	Cross-sectional
Yaakoubi et al (38), 2026	Tunisia	14–16	960	Cross-sectional
Zink et al (39), 2024	US	9–11	10,544	Longitudinal (prospective cohort)

The quality assessment showed that most of the studies were of moderate to high methodologic quality, with most between 6 and 9 stars on the NOS. Among cross-sectional studies, 13 studies received stars for representativeness and control of confounding. However, several studies were limited by incomplete reporting of response rates (n = 9) or small sample sizes (n = 4). Among longitudinal studies, most demonstrated adequate cohort selection, adjustment for confounding, and sufficient follow-up duration (n = 7). Nevertheless, ascertainment of screen time exposure was frequently based on self-reported or proxy-reported measures (n = 6). In particular, all but one of the longitudinal studies relied on self-reported or proxy-reported assessments of screen time. Notably, studies that formally tested interactions between screen time and sex were generally of high methodologic quality, with 8 stars in all but 1 study.

Most studies used self-reported measures of screen time (n = 17), often capturing multiple modalities. Among these, some studies aggregated different modalities into a composite measure of total screen time (n = 3), whereas others assessed each modality separately without combining durations (n = 16), referred to as mixed-itemized measures (Table 2). A small number of studies evaluated a single type of screen time exposure (n = 3). Screen time was analyzed by using either categorical cutoffs such as time thresholds (eg, ≥2 hours per day) or continuous measures.

**Table 2:** Screen Time Exposures and Obesity-Related Outcomes in Studies of Children, Adolescents, and Young Adults, 2015–2026

Author	Exposure definition (screen time)			Outcome definition (obesity)		
	Measurement	Content	Assessment	Measurement	Content	Assessment
Al-Hazzaa and Albawardi (18), 2019	Self-reported screen time	Mixed-itemized (television, games, computer)	Categorical (cut-off: ≤3 h/d vs >3 h/d)	Measured	Weight status classification (obesity level), central obesity (waist-to-height ratio)	Categorical (obese vs nonobese, waist-to-height ratio cutoff)^a^
Al-Shoaibi et al (19), 2026	Self-reported screen time	Mixed-itemized (television, videos, games, phones, social media)	Continuous, categorical (cut-off: 0–4 h/d, >4–8 h/d, >8 h/d)	Measured	Weight status classification (transition to normal BMI percentile)	Derived trajectories (transition to normal)^b^
Cai et al (20), 2022	Self-reported screen time	Total screen time (aggregate)	Categorical (cut-off: ≤2 h/d vs >2 h/d)	Measured	Obesity phenotypes (classified according to nutrition, metabolic status and BMI)	Categorical (metabolically healthy vs unhealthy obesity phenotypes)^c^
Domoff et al (21), 2021	Diary/recall-based	Specific screen type (television)	Continuous	Measured	BMI-based adiposity (BMI percentile)	Continuous (percentile)^d^
Foubister et al (22), 2023	Self-reported screen time	Specific screen type (social media)	Categorical (cut-off: 0–<1 h/d, 1–<3 h/d, 3–<5 h/d, ≥5 h/d)	Measured	BMI-based adiposity (BMI *z* score)	Continuous (*z* score)^e^
Furthner et al (23), 2018	Self-reported screen time	Mixed-itemized (television, video games, computers)	Categorical (cut-off: <2 h/d vs >2 h/d)	Measured	Weight status classification (overweight/obesity)	Categorical (percentile cutoffs: 85th, 95th)^b^
Golshevsky et al (24), 2020	Self-reported and proxy-reported (parent) screen time	Mixed-itemized (television, computers, video games)	Continuous	Clinical records	BMI-based adiposity (BMI, BMI *z* score), weight status classification	Continuous (*z* score), categorical (percentile cutoff: >95th)^b^
Kenney and Gortmaker (25), 2017	Self-reported screen time	Mixed-itemized (television, tablets, videogames, computers, smartphones)	Categorical (cut-off: 0, <1 h/d, 1 h/d, 2 h/d, 3 h/d, 4 h/d, ≥5 h/d)	Self-reported	Weight status classification (obesity)	Categorical (percentile cutoff: ≥95th)^b^
Lu et al (26), 2025	Self-reported screen time	Mixed-itemized (television, computer, mobile electronic devices)	Continuous, categorical (cut-off: 0, <1 h/d, 1–<2 h/d, ≥2 h/d)	Measured	BMI-based adiposity (BMI), weight status classification (incident overweight/obesity)	Continuous (BMI), categorical (incident overweight/obesity)^f^
O’Brien et al (27), 2018	Self-reported screen time	Mixed-itemized (television viewing, playing video games, computer usage)	Continuous, frequency-based, categorical (cut-off: ≤2 h/d vs >2 h/d to meet guidelines)	Measured	BMI-based adiposity (BMI), weight status classification	Continuous (BMI), categorical (normal, overweight, obese)^a^
Padmapriya et al (28), 2019	Proxy-reported screen time (parent/caregiver)	Mixed-itemized (television viewing/playing, computer, handheld devices)	Continuous	Measured	BMI-based adiposity (BMI), body composition (skinfolds)	Continuous (BMI, sum of skinfolds)
Parkes et al (29), 2020	Proxy-reported screen time (parent/caregiver)	Mixed-itemized (television, computers, games, mobile telephones)	Trajectory/pattern-based, categorical (cut-off: <2 h/d, 2–<4 h/d, ≥4 h/d)	Measured	Weight status classification (overweight/obesity)	Derived trajectories (obesity trajectory: eg, high/increasing obesity)^a^
Ren et al (30), 2017	Proxy-reported screen time (parent/caregiver)	Mixed-itemized (television, computer games)	Continuous	Parent-reported	BMI-based adiposity (BMI *z* score), weight status classification (overweight/obesity)	Continuous (*z* ^,^ g score), categorical (overweight, obese)^c^
Saelee et al (31), 2020	Self-reported screen time	Specific screen type (television)	Categorical (cut-off: <2 h/d vs ≥2 h/d)	Measured	Weight status classification (obesity)	Categorical (percentile cutoff: ≥95th)^b^
Sampasa-Kanyinga et al (32), 2020	Self-reported screen time	Total screen time (aggregate) and specific screen type (social media)	Categorical (cut-off: infrequent/none, moderate ≤2 h/d, heavy >2 h/d)	Self-reported	BMI-based adiposity (BMI *z* score)	Continuous (*z* score)^c^
Seral-Cortes et al (33), 2021	Self-reported screen time	Mixed-itemized (television viewing, computer games, video games, internet)	Continuous	Measured	BMI-based adiposity (BMI), body composition (fat mass index), central obesity (waist circumference)	Continuous^a^
Shakir et al (34), 2018	Diary/recall-based	Mixed-itemized (television, computer, video games)	Continuous	Measured	Body composition (DXA-based percentage of body fat), central obesity (waist-to-height ratio), weight status classification	Continuous (percentage body fat, waist-to-height ratio), categorical (weight status)^a^
Sun et al (35), 2021	Self-reported screen time	Mixed-itemized (television, video games, videotapes, videos)	Continuous	Measured	Weight status classification (overweight/obesity), central obesity (waist-to-height ratio)	Categorical (overweight/obesity, waist-to-height ratio cutoff)^g^
Tan et al (36), 2025	Self-reported screen time	Mixed-itemized (television, video games, computers)	Continuous	Measured	BMI-based adiposity (BMI *z* score), weight status classification (underweight, normal, overweight, obesity)	Continuous (*z* ^,^ f score), categorical (weight status)^c^
Xue et al (37), 2016	Self-reported and proxy-reported screen time (parent/caregiver)	Mixed-itemized (television viewing, computer use)	Continuous, categorical (cut-off: <2 h/d vs ≥2 h/d)	Measured	Body composition (percentage body fat, fat mass index, fat-free mass index), BMI-based adiposity (BMI SD score), weight status (overweight)	Continuous (log-transformed), categorical^g^
Yaakoubi et al (38), 2026	Self-reported and objectively measured screen time (device tracking)	Total screen time (aggregate) and specific screen type (smartphone)	Continuous (screen time), categorical (cut-off for problematic smartphone use)	Clinical records (school health records)	BMI-based adiposity (BMI *z* score), weight status classification (overweight/obesity)	Continuous (*z* score), categorical (*z* score cutoffs: >+1 SD, >+2 SD)^c^
Zink et al (39), 2024	Self-reported screen time	Mixed-itemized (streaming, gaming, socializing)	Continuous (modeled continuously as 30-min substitutions/proportions)	Measured	BMI-based adiposity (BMI *z* score)	Continuous (*z* score)^b^

Abbreviations: BMI, body mass index; DXA, dual-energy x-ray absorptiometry.

a International Obesity Task Force (40).

b US Centers for Disease Control and Prevention growth charts (41).

c World Health Organization (42).

d US Centers for Disease Control and Prevention BMI calculator (43).

e UK 1990 growth centiles (44).

f Chinese National Standard (45).

g Working Group on Obesity in China (46).

Obesity-related outcomes were assessed by using BMI-based indicators, including BMI and BMI *z* scores (n = 14), and weight status classifications (n = 12), with some studies incorporating both approaches (n = 10). Additionally, several studies included measures of central obesity and other indicators such as waist-to-height ratio and fat mass index (n = 8). The most commonly used reference standard was the US Centers for Disease Control and Prevention growth charts (n = 8), followed by the World Health Organization (n = 5) and International Obesity Task Force (n = 5).

Eighteen studies reported a significant positive association between screen time and obesity-related outcomes, whereas 4 studies reported no significant association (Table 3). Among studies reporting significant associations, 10 studies found associations in both sexes and 8 studies reported association observed only among females or males. Specifically, screen time of 2 hours per day was positively associated with obesity among both males and females (23), and 2 or more hours per day was positively associated with obesity among females (31). A dose–response relationship was also observed with greater total screen exposure across multiple screening modalities (eg, television, computer, mobile device use) associated with higher obesity risk in both sexes (25,26). Increased screen time was also positively correlated with fat mass markers (34,37). Longitudinal evidence further indicated that higher screen exposure was associated with unfavorable obesity trajectories over time (29).

**Table 3:** Screen Time–Obesity Associations and Sex-Related Patterns in Studies of Children, Adolescents, and Young Adults, 2015–2026

Study	Association with obesity	Sex-specific association	Test for interaction		Summary of findings
			Yes/no	*P* value	
Al-Hazzaa and Albawardi (18), 2019	Not significant	No association	Yes	.43	Screen time was not significantly associated with obesity in either males or females
Al-Shoaibi et al (19), 2026	Not significant	No association	Yes	.77	Screen time was not significantly associated with the likelihood of transitioning from overweight/obesity to a normal BMI in both males (HR = 0.99; 95% CI, 0.95–1.02) and females (HR = 1.00; 95% CI, 0.95–1.05)
Cai et al (20), 2022	Significant	Female-specific (significant interaction)	Yes	<.05	Excessive screen time of >2 h/d significantly increased the risk of metabolically unhealthy obesity in females only (females OR = 1.47; 95% CI, 1.06–2.04; males OR = 0.97; 95% CI, 0.79–1.20)
Domoff et al (21), 2021	Not significant	No association	No	NA	No significant correlation was found between television viewing and BMI percentile in either males (Pearson correlations, *r *= −0.12; *P* > .05) or females (Pearson correlations, *r* = −0.05; *P* > .05)
Foubister et al (22), 2023	Significant	Association observed only among females	No	NA	Using social media for ≥5 h/d was positively associated with higher BMI *z* scores in females (β = 0.17; 95% CI, 0.05–0.29) but not in males (β = 0.08; 95% CI, −0.06 to 0.22)
Furthner et al (23), 2018	Significant	Associations observed in both sexes	No	NA	Weekend screen time and watching television, playing video games, or using computers for >2 h/d were significantly associated with higher BMI
Golshevsky et al (24), 2020	Significant	Association observed only among females	No	NA	Increased screen time was associated with higher BMI primarily in females (β = 0.079; *P* < .001), with a stronger effect observed among those with inadequate sleep
Kenney and Gortmaker (25), 2017	Significant	Associations observed in both sexes	No	NA	Screen time ≥5 h/d significantly increased the odds of obesity in a dose–response pattern (females AOR = 1.85; 95% CI, 1.33–2.57; males AOR = 1.73; 95% CI, 1.26–2.37)
Lu et al (26), 2025	Significant	Both, stronger association in females (significant interaction)	Yes	.04	Screen-related sedentary time per hour increment was positively associated with BMI in both sexes, but the association was stronger in females (females β = 0.04; 95% CI, 0.02–0.05; males β = 0.02; 95% CI, 0.01–0.03 [*P* > .05])
O’Brien et al (27), 2018	Not significant	No association	No	NA	Screen time did not significantly predict BMI for either sex (males *r* = 0.087; females *r* = 0.034; *P* > .05). Playing computer games had a weak positive correlation with BMI in males only (*r* = 0.201; *P* < .05)
Padmapriya et al (28), 2019	Significant	Association observed only among males	No	NA	Screen time at 2–3 years of age was significantly associated with an increase in BMI and skinfold thickness at 3–5 years in males (males β = 0.18; 95% CI, 0.06–0.30; females β = −0.02; 95% CI, −0.14 to 0.09)
Parkes et al (29), 2020	Significant	Associations observed in both sexes (interaction not significant)	Yes	NA	Having an early increase trajectory of screens in the bedroom was significantly associated with higher risk of belonging to the high/increasing obesity trajectory (RRR = 2.55; 95% CI, 1.30–5.00) and higher risk of being in the high/stable overweight trajectory (RRR = 1.62; 95% CI, 1.01–2.57)
Ren et al (30), 2017	Significant	Association observed only among females	No	NA	Screen time had a significant positive association with BMI *z* scores in females but not males (females β = 0.09; *P* = .05; males β = 0.04; *P* > .05)
Saelee et al (31), 2020	Significant	Associations observed only among females	No	NA	Watching television for ≥2 h/wk was significantly associated with obesity in both males and females (females β = 0.122; *P* = .001; males β = 0.066; *P* = .059)
Sampasa-Kanyinga et al (32), 2020	Significant	Male-specific (significant interaction)	Yes	<.05	Heavy social media use of >2 h/d was significantly associated with higher BMI *z* scores in males only (males β = 0.323; 95% CI, 0.094–0.551; females β = 0.036; 95% CI, −0.292 to 0.364)
Seral-Cortes et al (33), 2021	Significant	Association observed only among females	No	NA	Screen time significantly moderated the beneficial association between the Mediterranean Diet and BMI/adiposity in females, but not in males. Interaction *P* value between screen time and Mediterranean Diet on BMI: females *P* = .02; males *P* = .06
Shakir et al (34), 2018	Significant	Associations observed in both sexes (television); association observed only among males (video game use)	No	NA	Television viewing was positively correlated with percentage body fat in both sexes, but video game use was positively associated with percentage body fat in boys only
Sun et al (35), 2021	Significant	Associations observed in both sexes (general obesity); association observed only among males (central obesity)	No	NA	Increases in screen time significantly raised the risk of obesity in both sexes (males OR = 1.15; 95% CI, 1.05–1.25; females OR = 1.12; 95% CI, 1.00–1.24) and central obesity in males (OR = 1.06; 95% CI, 1.02–1.10)
Tan et al (36), 2025	Significant	Associations observed in both sexes	No	NA	Screen-based sedentary behavior was positively associated with BMI, with weight concern acting as a mediator, and the strength of this mediation differing by sex. A significant 3-way interaction (sex × weight concern × overestimated body image) was observed (β = 0.08; *P* = .02). When body image perception was accurate, the indirect effect of screen time on BMI was stronger in males (males β = 0.055; 95% CI, 0.003–0.128; females β = 0.031; 95% CI, 0.002–0.066)
Xue et al (37), 2016	Significant	Associations observed in both sexes (fat-free mass index); association observed only among females (percentage body fat, fat mass index)	No	NA	Screen time was positively associated with fat-free mass index in both males (*P* for trend = .003) and females (*P* for trend <.001), whereas screen time’s positive association with direct fat mass indicators (percentage body fat, fat mass index) was significant in females only. Among males, the association of screen time with fat mass index and percentage body fat was no longer significant after further adjustment for energy intake.
Yaakoubi et al (38), 2026	Significant	Both, stronger association in males (significant interaction)	Yes	.01	Obesity significantly predicted excessive screen time and problematic smartphone use in both sexes, but the OR was higher for males (males OR = 5.5; 95% CI, 3.2–9.4; females OR = 3.8; 95% CI, 2.1–6.9)
Zink et al (39), 2024	Significant	Associations observed in both sexes, with different patterns	No	NA	Substituting screen time with physical activity lowered BMI in females, whereas substituting screen time with sleep lowered BMI in males.

Abbreviations: AOR, adjusted odds ratio; BMI, body mass index; HR, hazard ratio; NA, not applicable; OR, odds ratio; RRR, relative risk ratio.

Six studies reported associations observed only among females, whereas 4 studies reported associations observed only among males. Among the studies reporting associations observed only among females, 1 study found that increased screen time was significantly associated with higher percentage body fat and fat mass index, even after adjusting for energy intake (37). Excessive total screen time (>2 hours per day) was also associated with a 1.47-fold increased risk of metabolically unhealthy obesity among girls only (20). In addition, total screen time across multiple screen modalities significantly moderated the association between the healthy diet and BMI/adiposity among females but not among males (33) and substituting screen time with physical activity was associated with lower BMI only among females (39). Psychologic factors also appeared relevant, as the combination of overestimated body size and high levels of concern about weight was associated with increased BMI among girls (36).

In contrast, associations observed only among males were reported in a smaller number of studies (n = 4). Two studies reported that video game use was associated with obesity among boys only (27,34). Other studies found that obesity was more strongly associated with problematic smartphone use (38) and heavy social media use (32) among boys compared with girls. In some studies, associations between screen time and fat mass indicators among males were attenuated after adjustment for energy intake (37). Additionally, substituting screen time with sleep was associated with lower BMI among males (39). Formal tests for interaction were conducted in 7 studies (18–20,26,29,32,38), of which 4 reported significant interaction effects between sex and screen time (20,26,32,38).

## Discussion

This review identified both shared and potentially sex-specific patterns in the association between screen time and obesity-related outcomes. Findings are consistent with evidence from studies in adult populations (10–12), which have proposed several mechanisms that may explain this association, including reduced energy expenditure, increased energy intake through snacking during screen use, greater exposure to food advertising, and poor sleep quality. These behaviors often cluster, potentially strengthening their effect on weight gain (10–12,40). Similar mechanisms may also contribute to the association between screen time and obesity among children and adolescents.

Although evidence from adult populations may not be directly generalizable to children and adolescents, screen time may have greater implications for obesity risk in this younger population due to several factors. Because children and adolescents are in a developmental stage in which physical activity plays a central role, increased screen time may lead to greater reduction in energy expenditure and more pronounced disruptions in healthy weight regulation (19,22,23). They are also in a stage of developing dietary habits, during which screen-related behaviors — such as increased snacking during screen use, exposure to food advertising, and patterns of mindless eating — may be more readily established and persist into adulthood (20,24–26,30,37). They may also be particularly susceptible to food advertising, especially within contemporary digital environments where commercial messages are embedded in entertainment content and amplified by influencer-driven marketing (36). Furthermore, the effects of screen time on sleep may be more substantial in this population than in the adult population, given developmental changes in circadian rhythms and heightened vulnerability of sleep-related hormonal systems (19,20,31,32). Sleep disruption associated with screen use may affect appetite-regulating hormones such as leptin, leading to increased appetite and consequent weight gain (20,32). These physiological and behavioral mechanisms, along with the potential for early-life exposures to shape long-term obesity trajectories, underscore the importance of proactive management of screen time among children and adolescents.

This review identified evidence suggestive of sex differences in the association between screen time and obesity-related outcomes, indicating both shared and sex-specific underlying mechanisms (Figure 2). From a physiological and metabolic perspective, stronger associations between screen time and adiposity have been observed among females (20,22,24,30,33,37), including increases in fat mass indicators (37), a stronger dose–response relationship (26), and an increased risk of metabolically unhealthy obesity (20). In contrast, among males, associations were more often nonsignificant (20,22,24,30,33) or became nonsignificant after adjustment for energy intake (37), suggesting that screen time may act primarily as a behavioral driver of increased energy intake rather than as an independent determinant of adiposity. These observed patterns may reflect sex-specific variations in body composition and developmental trajectories, which have been proposed to influence endocrine and metabolic profiles (26,37,39).

**Figure 2:**
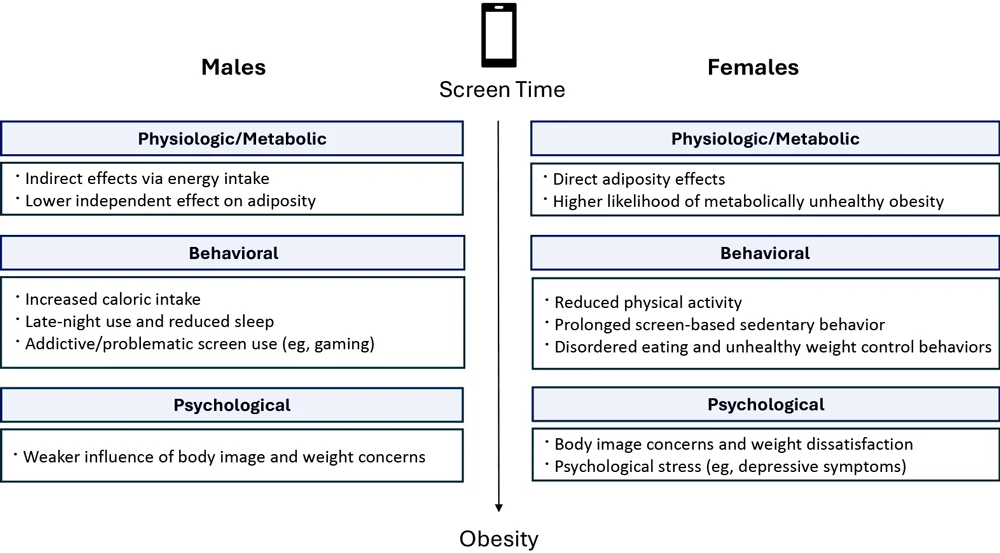
Conceptual model for sex-specific pathways linking screen time to obesity among children, adolescents, and young adults.

Sex differences in the association between screen time and obesity may also be explained through behavioral pathways. Evidence suggests that addictive eating behaviors associated with screen use may be more pronounced among males. In several studies, the association between screen time and fat mass was no longer significant after adjustment for energy intake, suggesting that increased caloric intake during screen use may partially explain the observed association between screen time and obesity (21,37). In contrast, among females, screen time remained positively associated with body composition indicators (percentage of body fat, fat mass index, fat-free mass index) even after adjustment for energy intake (33,37), suggesting that the observed association may not be fully explained by energy intake alone. This pattern among males may, in part, be explained by high-calorie snacking during screen use (26,28) and unmindful overeating due to disrupted satiety regulation (29,34). In addition, exposure to marketing of energy-dense foods and beverages (eg, fast food, sugar-sweetened beverages, salty snacks) through screen media may further increase total energy intake by promoting unhealthy dietary choices (18,21).

Sleep has been suggested as a potential behavioral pathway linking screen time and obesity. Screen time, particularly late-night social media use and device use in the bedroom, has been associated with delayed sleep onset (29). Several studies suggest that males may be more likely to engage in screen use that directly interferes with sleep, such as using smartphones or gaming while in bed (29,34), as well as problematic smartphone use (38) and heavy social media use (32). Additionally, substituting screen time with sleep has been associated with lower BMI among males but not among females (24,39). Screen use before bedtime may delay sleep onset by increasing cognitive arousal and through exposure to blue light, which suppresses melatonin secretion and impairs sleep quality (24,31,32,38). Sleep deprivation may contribute to obesity through multiple mechanisms, including increased ghrelin and decreased leptin levels, leading to increased appetite, as well as metabolic dysregulation characterized by insulin resistance, which may promote fat accumulation (20,24,25,31,32,39). Delayed sleep onset may also increase the likelihood of late-night eating and preference for energy-dense foods (24,32). Among behavioral factors, physical activity appears to have a particularly complex relationship with screen time and obesity which may also differ by sex. Substituting screen time with physical activity has been associated with BMI reductions among females but not among males (39), and longer television viewing has been linked to lower physical activity levels among females but not among males (25). Conversely, other studies report that screen time remains independently associated with increased adiposity even after adjusting for physical activity (32,34,37). A commonly proposed mechanism is the time-displacement hypothesis, which suggests that screen-based activities may replace time available for physical activity and consequently reduce energy expenditure. However, several studies suggest that the relationship between screen time and physical activity may differ by sex (27,28,38,39). One possible explanation is that non–screen time may be more likely to involve physical activity among males, whereas among females it may still be occupied by other sedentary behaviors, suggesting that reductions in screen time may not necessarily translate into equivalent increases in physical activity across sexes. Taken together, these findings suggest that potential sex differences in the screen time–obesity relationship may extend beyond metabolic and physiologic factors. Behavioral patterns related to diet, sleep, and physical activity may contribute to heterogeneity in the observed associations between screen time and obesity.

Psychologic factors may be an important but understudied pathway linking screen time and obesity, with some evidence suggesting potential differences by sex. Excessive screen time, particularly social media use, has been associated with increased weight concern among females, which may contribute to disordered eating, depressive symptoms, and unhealthy weight-control behaviors (22,36). Girls are more likely than boys to have negative body image concerns, which are associated with a higher likelihood of engaging in extreme weight-related behaviors (36,39). With the widespread use of digital editing and enhancement tools, adolescents are increasingly exposed to unrealistic beauty standards, which may contribute to body weight dissatisfaction and lower self-esteem. These factors have been suggested to be associated with disordered eating, depressive symptoms, and obesity-related outcomes (36,39).

This review has public health implications. The consistently observed association between screen time and obesity-related outcomes suggests that excessive screen exposure may be an important factor to consider in childhood obesity prevention efforts. The findings also suggest that screen time may be associated with obesity-related outcomes through multiple behavioral and psychosocial pathways that could differ by sex. These findings suggest that obesity prevention strategies may benefit from considering both the amount and context of screen use, as well as potential sex-specific pathways linking screen time to obesity-related outcomes. These strategies may involve moving beyond simply advising children and adolescents to reduce screen time and considering how non–screen time is structured and used. For example, because available evidence suggests that physical activity may play a more prominent role in the association between screen time and obesity among girls, interventions promoting physical activity and active alternatives to screen use may be particularly relevant for girls. For boys, problematic screen use, screen-associated eating behaviors, and sleep-related factors may be particularly relevant pathways, suggesting potential targets for intervention. Initial practical steps could include routine assessment of screen use patterns, sleep behaviors, physical activity, and screen-related psychosocial factors, with interventions tailored to individual risk profiles and needs. Particular attention could be given to screen-influenced psychological factors that may differentially shape obesity risk across sexes. At the population level, these findings suggest that integrated, multicomponent interventions across family, school, and digital environments, including regulation of food marketing, school-based screen use policies, and scalable digital health interventions may be beneficial. Despite these potential implications, further research is needed to determine the most effective strategies and the extent to which interventions should be tailored by sex and behavioral context.

### Limitations

This review has several limitations. The small number of eligible studies reflects the limited evidence base on the association between screen time and obesity in children, adolescents, and young adults. In addition, heterogeneity in exposure and outcome definitions and differences in study populations and designs constrain the current evidence base. Most studies relied on self-reported or proxy-reported measures of screen time, which may be subject to recall and reporting bias and could influence the observed associations. The predominance of cross-sectional study designs also limits causal inference. Finally, because this review was restricted to studies reporting sex-specific effect estimates or formal tests of interaction, the available evidence may be subject to reporting bias and may not fully capture studies that found no evidence of sex differences. Although the included studies were generally of moderate to high methodologic quality, evidence remains inconclusive because of such methodologic inconsistencies. Further research using standardized measures of screen time and obesity-related outcomes, formal tests of interaction, and longitudinal designs is warranted to better understand potential sex-specific pathways and inform targeted interventions.

Despite these limitations, a key strength of this review is its focus on sex-specific patterns in the association between screen time and obesity-related outcomes, an area that remains relatively underexplored in the literature but is of public health relevance. Because screen use is likely to continue increasing, driven by the rapid expansion of digital technologies, mobile devices, and online environments, a growing proportion of children and adolescents will be exposed to screen-based behaviors that have been associated with obesity-related outcomes. By synthesizing evidence across diverse settings and screen modalities, this study extends current understanding of the relationship between screen time and obesity, highlighting the importance of considering potential sex-related differences in future research and intervention development.

## Appendix Search Strategy for a Systematic Review of Screen Time and Obesity Among Children and Adolescents With a Focus on Sex-Specific Differences

**PubMed**

(("Screen Time"[Mesh] OR "Television"[Mesh] OR "Video Games"[Mesh] OR "Smartphone"[Mesh] OR "Social Media"[Mesh]) OR (screen time[tiab] OR television[tiab] OR "TV viewing"[tiab] OR smartphone*[tiab] OR "mobile phone*"[tiab] OR "social media"[tiab] OR "video game*"[tiab] OR gaming[tiab])) AND ("Obesity"[Mesh] OR "Pediatric Obesity"[Mesh] OR "Overweight"[Mesh] OR obes*[tiab] OR overweight[tiab] OR BMI[tiab] OR "body mass index"[tiab] OR adiposity[tiab] OR "body fat"[tiab] OR "fat mass"[tiab]) AND ("Child"[Mesh] OR "Adolescent"[Mesh] OR child*[tiab] OR adolescen*[tiab]) AND ("sex differences"[tiab] OR "gender differences"[tiab] OR male[tiab] OR female[tiab] OR interaction[tiab] OR "effect modification"[tiab]) NOT("self-esteem"[tiab] OR "self esteem"[tiab] OR "self-concept"[tiab] OR "self concept"[tiab] OR "self-perception"[tiab] OR "self perception"[tiab]) NOT ("Adult"[Mesh] NOT ("Child"[Mesh] OR "Adolescent"[Mesh])) NOT (animals[MeSH Terms] NOT humans[MeSH Terms]) AND ("2016/01/01"[Date - Publication] : "3000"[Date - Publication])

**CINAHL**

((MH "Screen Time" OR MH "Television Viewing" OR MH "Video Games" OR MH "Smartphones" OR MH "Social Media") OR (TI (screen time OR television OR "TV viewing" OR smartphone* OR "mobile phone*" OR "social media" OR "video game*" OR gaming) OR AB (screen time OR television OR "TV viewing" OR smartphone* OR "mobile phone*" OR "social media" OR "video game*" OR gaming))) AND ((MH "Obesity+" OR MH "Overweight") OR (TI (obes* OR overweight OR BMI OR "body mass index" OR adiposity OR "body fat" OR "fat mass") OR AB (obes* OR overweight OR BMI OR "body mass index" OR adiposity OR "body fat" OR "fat mass"))) AND ((MH "Child+" OR MH "Adolescence")) AND ((TI ("sex differences" OR "gender differences") OR AB ("sex differences" OR "gender differences"))) NOT (AB ("self-esteem" OR "self esteem")) Limiters: Published Date: 20160101-20261231

**Embase**

(('screen time'/exp OR 'television'/exp OR 'video game'/exp OR 'smartphone'/exp OR 'social media'/exp) OR ('screen time':ti,ab OR television:ti,ab OR 'tv viewing':ti,ab OR smartphone*:ti,ab OR 'mobile phone*':ti,ab OR 'social media':ti,ab OR 'video game*':ti,ab OR gaming:ti,ab)) AND (('obesity'/exp OR 'pediatric obesity'/exp OR 'overweight'/exp) OR (obes*:ti,ab OR overweight:ti,ab OR bmi:ti,ab OR 'body mass index':ti,ab OR adiposity:ti,ab OR 'body fat':ti,ab OR 'fat mass':ti,ab)) AND (('child'/exp OR 'adolescent'/exp) OR (child*:ti,ab OR adolescen*:ti,ab)) AND ('sex differences':ti,ab OR 'gender differences':ti,ab OR male:ti,ab OR female:ti,ab OR interaction:ti,ab OR 'effect modification':ti,ab) NOT ('self esteem'/exp OR 'self esteem':ab OR 'self-esteem':ab OR 'self concept':ab OR 'self-concept':ab OR 'self perception':ab OR 'self-perception':ab) NOT ('adult'/exp NOT ('child'/exp OR 'adolescent'/exp)) NOT ('animal'/exp NOT 'human'/exp) AND [2016-2026]/py
